# Landscape of metabolic alterations and treatment strategies in breast cancer

**DOI:** 10.1016/j.gendis.2025.101521

**Published:** 2025-01-08

**Authors:** Xiujuan Wu, Xuanni Tan, Yangqiu Bao, Wenting Yan, Yi Zhang

**Affiliations:** Department of Breast and Thyroid Surgery, Southwest Hospital, Army Medical University, Key Laboratory of Chongqing Health Commission for Minimally Invasive and Precise Diagnosis and Treatment of Breast Cancer, Chongqing 400038, China

**Keywords:** Amino acid metabolism, Breast cancer, Glucose metabolism, Lipid metabolism, Metabolic alterations

## Abstract

Breast cancer, the most prevalent cancer in women, poses a significant threat to their health. One of the prominent characteristics of malignant transformation in breast cancer cells is metabolic reprogramming, which encompasses glucose, lipid, and amino acid metabolism. Notably, breast cancer cells exhibit augmented energy metabolism and heightened glycolysis. In addition, there is an escalated demand for glutamine, which is met through intrinsic synthesis, uptake from extracellular sources via membrane transport proteins, or up-regulation of key metabolic enzymes in the glutamine metabolism pathway. Lipids not only serve as an energy source for tumor cells but also function as signaling molecules for intercellular communication. Extensive research in recent years has focused on unraveling the intricate mechanisms underlying metabolic reprogramming. Consequently, genes implicated in these processes have emerged as clinical therapeutic targets for cancer treatment. This review provides a comprehensive summary of the common metabolic alterations observed in cancer cells, discusses the factors and regulatory mechanisms influencing these changes, and explores potential therapeutic targets and strategies within the realm of cancer metabolism.

## Introduction

The incidence of new cases of breast cancer (BC) has shown a consistent increase over the past four decades, with projections indicating continued growth.^1^, ^2^, ^3^ BC is the most prevalent cancer among females across the globe, accounting for approximately 30 % of all female cancers, with a mortality-to-incidence ratio of 15 %.^4^ The incidence rate of BC varies across different regions, reflecting the interplay between economic development, social factors, and lifestyle choices.^5^ Factors contributing to the rising incidence rate include extended life expectancy, enhanced screening practices, delayed childbearing, and obesity.^6^^,^^7^ In contrast, the overall mortality rate associated with BC is declining. The demise of patients with BC is significantly associated with metastatic cancer progression. With the onset of distant metastasis, the 5-year survival rate has plummeted from 99 % to 27 %.^8^

The risk factors associated with BC primarily encompass genetic mutations. Approximately 10 % of cancer cases are attributable to genetic predisposition. The most prevalent BC-related hereditary mutations are found in BRCA1 and BRCA2 genes, with an average cumulative lifetime risk of approximately 70 %.^9^ The significant prevalence of BC can be attributed to diverse factors, such as pregnancy-related influences, hormone therapy, and lifestyle (*e.g*., obesity, alcohol consumption, low-fiber diet, and smoking).^10^

BC exhibits significant cellular and molecular heterogeneity, as evidenced by the expression patterns of estrogen receptor (ER), progesterone receptor (PR), and human epidermal growth factor-2 (HER2). This heterogeneity leads to the classification of BC into four distinct subtypes: luminal A (ER^+^ and/or PR^+^, HER2^−^), luminal B (ER^+^ and/or PR^+^, HER2^+^), HER2 (HER2^+^, ER^−^, PR^−^), and triple-negative (ER^−^, PR^−^, HER2^−^). Among these, triple-negative BC (TNBC) represents a high-risk basal subtype, accounting for approximately 15 %–20 % of all BC cases.^11^ Patients with TNBC typically present with earlier onset at a young age, lower histological differentiation, unfavorable prognosis, increased recurrence and metastasis rates, and higher mortality. These characteristics have drawn considerable attention in both research and clinical settings in recent years. Notably, the absence of ER, PR, and HER2 expression in patients with TNBC limits their ability to benefit from existing endocrine therapies and HER2-targeted molecular treatments. At present, the primary treatment options for TNBC involve cytotoxic chemotherapy with drugs such as paclitaxel and anthracycline; however, this strategy is associated with limited efficacy.^12^

Metabolic alterations have emerged as a captivating avenue for cancer exploration and treatment, garnering substantial attention over the past decade. Dr. Otto Warburg was a pioneer in this field, being the first to detect metabolic changes in cancer cells. He observed that, unlike normal cells, cancer cells exhibit a preference for glycolysis even in the presence of oxygen-rich environments.^13^ Advancements in scientific research and technology have further corroborated the significance of metabolic reprogramming in various cancer types. The International Agency for Cancer Research has identified an association between excessive body fat and different cancer types, including postmenopausal BC.^14^ This association between obesity and BC is particularly strong in ER^+^ subtypes.^15^^,^^16^ Consequently, metabolic alterations hold the potential for targeted therapeutic interventions.

This review provides a comprehensive overview of the physiological and metabolic characteristics of various BC subtypes. We delve into the intricate mechanisms through which metabolic changes propel tumor initiation, sustain tumor growth, and govern tumor metastasis. Moreover, we explore the potential of modulating metabolism as a strategy to enhance the efficacy of anti-cancer treatments. These findings establish a robust foundation for the rational design of metabolic drugs targeting BC.

### Amino acid metabolism in BC, with emphasis on glutamine and its role in tumor proliferation

Amino acids are essential molecules for protein synthesis and play a key role in various cellular functions, including energy production, nucleoside synthesis, and cellular redox balance maintenance. They can be categorized as non-essential and essential amino acids. Essential amino acids must be acquired from exogenous sources as cells cannot synthesize them *de novo*. Tumor cells, which undergo rapid proliferation, have an increased demand for amino acids, which demands metabolic reprogramming to meet these nutritional requirements for accelerated growth.^17^^,^^18^ In the context of BC, recent studies have revealed a compelling association between the metabolic pathways of amino acids, specifically glutamine, serine, and glycine, and the proliferation and progression of tumors.^19^, ^20^, ^21^ Glutamine, in particular, has been extensively scrutinized. It is the most abundant amino acid in human plasma and serves as a vital substrate for carbon metabolism. In addition, glutamine functions as a nitrogen source, significantly contributing to nucleotide synthesis and nonessential amino acid generation.^22^ It also actively participates in glutathione synthesis and cellular stability maintenance in the presence of reactive oxygen species.^23^ Glutamine is classified as a “conditionally essential amino acid” in humans. While it can be obtained through dietary intake by digestion and absorption in the small intestine, it is also synthesized and secreted by glutamine synthetase within various tissues, including the lungs, skeletal muscles, and adipose tissue. Tumor cells, however, cannot meet their high-demand requirements for rapid proliferation solely through endogenous glutamine synthesis. To fulfill their demands, they rely on the uptake of extracellular glutamine via membrane transporters or up-regulate the expression and activity of pivotal metabolic enzymes within the glutamine metabolic pathway. Upon entering the cell, glutamine undergoes deamination by glutaminase in the mitochondria, resulting in glutamate production. Subsequently, glutamate dehydrogenase and transaminase act to generate α-ketoglutarate along with NADH/NADPH. α-Ketoglutarate and NADH/NADPH then enter the tricarboxylic acid cycle and play a crucial role in regulating intracellular redox homeostasis.^24^^,^^25^ The up-regulation of transaminase and glutamate dehydrogenase expression in BC cells is associated with the enhanced proliferative capacity of these cells.^26^ Notably, reducing the expression of these enzymes has been demonstrated to effectively inhibit BC cell proliferation. In addition, distinct amino acid metabolism characteristics are observable among different molecular subtypes of BC. For instance, ER^−^ BC cells exhibit heightened glutaminase expression, which is inversely correlated with patient prognosis.^27^ In ER^+^ luminal A BC, glutaminase and glutamate dehydrogenase expression are reportedly less pronounced, whereas in HER2^+^ BC, their expression is elevated.^20^ Furthermore, in comparison with ER^+^ BC, ER^−^ BC, including HER2^+^ BC and TNBC, exhibits increased glutamine uptake and enhanced glutamine catabolism. This heightened metabolic activity in ER^−^ BC can be attributed to increased levels of glutamic acid and decreased levels of glutamine.^28^^,^^29^

### Influence of oncogenes, tumor suppressor genes, and amino acid transporters on glutamine metabolism in BC

Multiple key aspects contribute to the regulation of glutamine metabolism in BC. This process is primarily influenced by the interplay of oncogenes and/or tumor suppressor genes; besides, the modulation of amino acid transporters plays a crucial role in this pathway, along with the activity of relevant enzymes. One noteworthy factor is c-Myc, a transcription factor that is frequently overexpressed in different cancer types, including BC, as identified through The Cancer Genome Atlas bioinformatics analysis and *in vitro* validation.^28^^,^^30^ Researchers have confirmed via *in vitro* experiments that c-Myc up-regulates the expression of the glutamine transporter SLC1A5, enhancing the uptake and utilization of glutamine. This is achieved by inhibiting miR-23a and miR-23a/b, which results in glutaminase 1 overexpression.^31^ Besides, analyzing clinical BC and normal tissue samples, along with a Gene Expression Omnibus microarray dataset, revealed that miR-513c regulated glutamine metabolism by down-regulating glutaminase. miR-513c overexpression may exert a significant suppressive effect on the progression of BC cells.^32^ Another crucial component in glutamine-related signaling pathways is glutamate, which activates various downstream signaling cascades through the N-methyl-d-aspartate receptor. Notably, activation of the guanylate-kinase-associated protein signaling pathway by glutamate promotes cancer cell invasion and induces glutamate secretion in TNBC.^33^ P53, a prominent tumor suppressor gene known as the “guardian of the genome”, plays a vital role in glutamine metabolism regulation. It does so by modulating the expression of numerous genes involved in cellular metabolism, including those implicated in glutamine metabolism. According to a review published in *Nature Reviews Cancer* in 2009, empirical investigations have demonstrated that P53 effectively inhibits the expression of glutaminase, consequently decreasing glutamine utilization by cancer cells.^34^ Furthermore, the tumor suppressor gene Rb regulates glutamine uptake. *In vivo* studies in mice and *in vitro* cell biology experiments have indicated that the Rb-controlled transcription factor E2F-3 modulates glutamine uptake by directly regulating ASCT2 mRNA and protein expression. The Rb/E2F cascade directly regulates a crucial energetic and anabolic pathway essential for neoplastic growth^35^ (Table 1).

**Table 1:** Regulation of glutamine in breast cancer cells.

Key factor	Function	Regulation/mechanism	Refs
c-Myc	Oncogenic	c-Myc upregulate the expression of the glutamine transporter SLC1A5, enhancing the uptake and utilization of glutamine	28,30,31
miR-23a miR-23a/b	Tumor suppressive	c-Myc can inhibit miR-23a and miR-23a/b, which results in GLS1 overexpression	31
miR-513c	Tumor suppressive	miR-513c gene in regulating glutamine metabolism by downregulating glutaminases	32
P53	Tumor suppressive	P53 effectively inhibits the expression of glutaminase, consequently decreasing glutamine utilization by cancer cells	34
Rb	Tumor suppressive	The Rb-controlled transcription factor E2F-3 evidently modulates glutamine uptake by directly regulating ASCT2 mRNA and protein expression	35
SLC1A5	Oncogenic	This upregulation of SLC1A5 expression results in a substantial increase in glutamine uptake mediated by SLC1A5	39,40

The transport of amino acids, including glutamine, requires specific transporters that facilitate their passage across the cell membrane. There are approximately 14 amino acid transporters dedicated to transporting glutamine, classified into four distinct solute carrier (SLC) families.^36^^,^^37^ The SLC1A5 subtype, in particular, has received considerable attention in the context of BC. Its expression exhibits variations depending on molecular subtypes. In-depth proteomic profiling analysis has substantiated that SLC1A5 serves as an independent prognostic factor for BC.^38^ Its elevated expression has been specifically observed in HER2-type BC and triple-negative basal-like BC cells. This up-regulation of SLC1A5 expression results in a substantial increase in glutamine uptake mediated by SLC1A5.^39^ In ER^−^ BC cells, tamoxifen and raloxifene have been observed to effectively suppress SLC1A5 expression, leading to restrained glutamine uptake and proliferation of these cancer cells.^40^ On the other hand, the up-regulation of SLC1A5 expression impairs the sensitivity of BC cells to paclitaxel.^41^ Moreover, SLC1A5 is implicated in promoting endocrine resistance in BC cells.^42^ Other subtypes of SLC transporters have also been investigated in the context of BC. SLC6A14 exhibits high expression levels in ER^+^ BC,^43^ and its mechanism of action may be linked to the mammalian target of rapamycin (mTOR) signaling pathway.^44^ Notably, SLC6A14 is targeted by miR-23a,^45^ and its regulation is influenced by c-Myc. It has also been observed that SLC7A5 is expressed at higher levels in HER2^+^ BC and patients with TNBC as compared with those with luminal-type BC.^46^ The expression of SLC7A11 is significantly up-regulated in ER^+^ BC and TNBC, leading to increased cysteine influx and glutathione synthesis.^47^^,^^48^ Moreover, SLC7A11 expression was reported to be up-regulated in a mouse model of brain metastasis.^49^ Several studies have found a correlation between the expression of SLC39 transporters and BC. In particular, SLC39A2, SLC39A3, SLC39A4, SLC39A5, SLC39A7, SLC39A12, and SLC39A13 exhibit high expression levels in BC and are associated with poor patient prognoses.^50^

### BC treatment: targeting glutamine metabolism for potential therapeutic strategies

**Figure 1:**
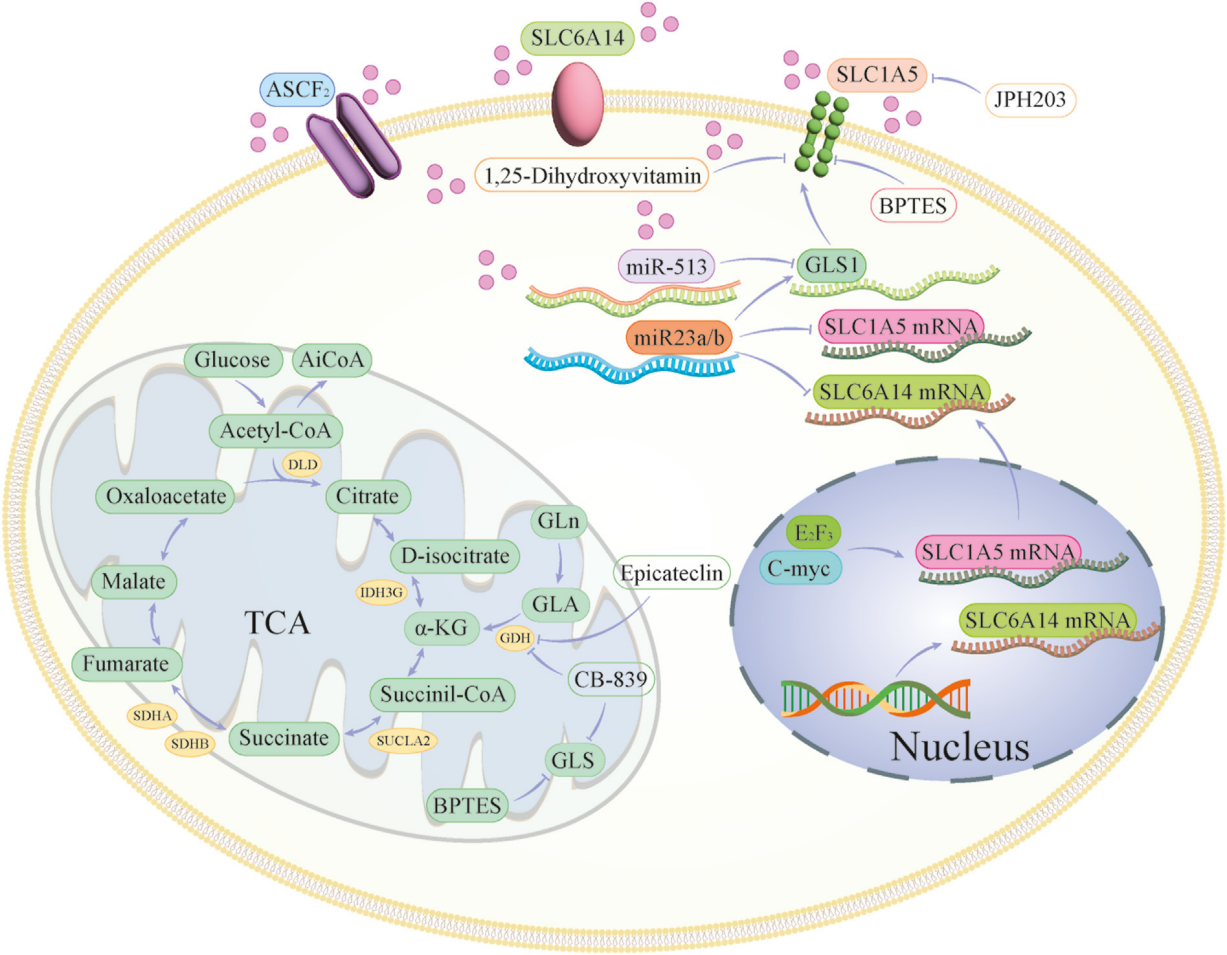
Reconstruction of amino acid metabolism in breast cancer (BC). Amino acid metabolism in BC has been extensively studied, with a focus on the role of various regulatory genes. Notably, genes such as c-Myc, miR-513c, and Rb have been identified as key regulators that influence the expression and activity of crucial metabolic enzymes in the glutamine metabolism pathway. In addition, they influence the function of amino acid transport proteins, considerably affecting BC cell proliferation and metastasis.

In the domain of BC treatment, extensive research has been conducted on targeted drugs aimed at modulating glutamine metabolism. This research area primarily focuses on glutamine metabolism regulation through diverse approaches, including inhibiting crucial enzymes within the glutamine metabolism pathway, suppressing glutamine uptake, and targeting pivotal targets within signaling pathways associated with glutamine metabolism products. Several inhibitors, specifically targeting glutaminase, have demonstrated significant anti-tumor efficacy. A noteworthy example is bis-2-(5-phenylacetamido-1,2,4-thiadiazol-2-yl) ethyl sulfide 3, a selective allosteric modulator of glutaminase 1. Experimental studies involving animal xenograft and transgenic mouse models with up-regulated c-Myc gene expression have reported that bis-2-(5-phenylacetamido-1,2,4-thiadiazol-2-yl) ethyl sulfide 3 effectively retards tumor growth.^51^^,^^52^ Telaglenastat (CB-839), a highly potent and selective inhibitor of glutaminase 1, is currently in phase I clinical trials. This compound effectively catalyzes the deamidation process, converting glutamine into glutamate. Its oral administration has been observed to exert anti-proliferative and anti-tumor effects, specifically in TNBC cell lines.^53^ Furthermore, epicatechin, a principal component found in green tea polyphenols, has been identified as an inhibitor of glutamate dehydrogenase, capable of inhibiting the growth of glutamine-dependent BC cells.^54^^,^^55^ Riluzole exerts inhibitory effects on the growth of TNBC by impeding the release of glutamate through the blockade of mGlu R1 receptor activation.^56^ JPH203, a selective inhibitor targeting SLC7A5, has shown substantial efficacy in inhibiting tumor cell growth across various human cell lines and mouse models.^57^ 1,25-dihydroxyvitamin D [1,25(OH)2D], the biologically active form of vitamin D, plays a significant role in down-regulating SLC1A5 expression. Consequently, it diminishes glutamine uptake and utilization by BC cells, contributing to BC prevention.^58^ Sulfasalazine, an inhibitor of SLC7A11, reportedly elevates the intracellular levels of glutamine while concurrently inhibiting the expression of mucin 1 (MUC1) in TNBC-specific cell lines.^59^ These findings hold promise for targeted therapy in modulating glutamine metabolism; however, it is crucial to emphasize that further research and comprehensive clinical trials are imperative to substantiate the effectiveness and safety of these approaches (Fig. 1).

### Lipid metabolism and its implications in BC treatment

In recent years, numerous studies have unveiled a strong relationship between metastasis, drug resistance, and lipid metabolism across various cancer types, including BC.^60^, ^61^, ^62^, ^63^ Delving into the intricate heterogeneity of lipid metabolism holds significant promise in identifying novel therapeutic targets for BC treatment. However, it is crucial to acknowledge that tumor lipid metabolism is influenced by diverse factors, encompassing both intracellular complexities and extracellular cues.^64^

### Fatty acid metabolism in cancer

Lipids play a crucial role in maintaining cellular homeostasis, serving as energy sources and functioning as signaling molecules to facilitate signal transduction processes. Current research suggests a significant association between aberrant lipid metabolism and tumor initiation and progression, which has piqued the interest of numerous investigators. Significant lipid metabolism alterations in BC predominantly involve enhanced fatty acid uptake, storage, *de novo* lipogenesis, and oxidative utilization to generate ATP.^65^

Mammalian cells employ two prominent pathways to acquire lipids: extracellular uptake and neoadipogenesis. *De novo* lipogenesis, in contrast, is predominantly observed in select cell types, such as adipocytes and hepatocytes.^66^ In cancer cells, augmented fatty acid uptake is commonly mediated through the up-regulation of fatty acid transport protein 1 (FATP1) and CD36 expression, while adipocytes and endothelial cells in the tumor microenvironment exhibit the expression of fatty acid-binding protein 4 (FABP4) to facilitate this process.^62^^,^^67^ A correlation reportedly exists between circulating FABPs derived from adipocytes in obese women and BC progression.^68^ Lipid droplets actively provide fatty acids via lipolysis and lipid phagocytosis of triacylglycerol, serving as a primary fuel source for mitochondrial oxidative metabolism during nutrient insufficiency.^69^ Furthermore, lipid droplets play a pivotal role in driving cancer progression by inducing epithelial–mesenchymal transition within the acidic tumor microenvironment.^70^ However, the molecular mechanisms underlying this phenomenon necessitate further elucidation. The generation of fatty acids establishes a direct relationship between lipid metabolism and glucose/glutamine metabolism. Key enzymes involved in this process, such as ATP-citrate lyase, acetyl-CoA carboxylase, and the multifunctional fatty acid synthase (FASN), are regulated by the transcription factor REBP1 and the mTOR signaling pathway in cancer cells.^71^ Heightened activation of *de novo* lipogenesis is essential for neoplastic cells to sustain their metabolic requirements.^72^ Increased FASN expression has been linked to BC progression as well as the development of resistance to anti-cancer therapeutics.^73^^,^^74^ Furthermore, compelling evidence suggests that low-density lipoprotein contributes to BC progression in both human and murine cellular models,^75^ although further investigations are warranted to comprehensively understand the underlying molecular mechanisms.

### The factors affecting lipid metabolism in BC

#### Intrinsic factors of heterogeneity

Cancer, arising from somatic cell evolution, results from the altered regulation of proto-oncogenes and tumor suppressor genes through genetic mutations.^76^ Gene sequence perturbations can disrupt downstream cellular pathways, including those involved in cell metabolism. Epigenetic mechanisms play a critical role in modulating gene expression by mediating the methylation and acetylation of histones and DNA via specific enzymatic activities, influencing the accessibility of transcription factors to target genes.^77^ Epigenetic modifications contribute to tumor heterogeneity. Epigenetic regulation is evidently capable of modulating lipid metabolism in BC cells. In these cells, several genes involved in lipid metabolism display heightened expression, thereby promoting *de novo* fatty acid synthesis. The dysregulation of these genes has been implicated in BC proliferation, metastasis, and resistance to therapeutic interventions.^60^^,^^61^^,^^78^

P53, a prominent tumor suppressor transcription factor, plays a fundamental role in maintaining genomic integrity, and its mutations are frequently implicated in various malignancies. A significant association exists between mutant P53 depletion and the restoration of a more orderly cellular morphology in BC cells within three-dimensional culture models. Furthermore, studies have revealed that P53 mutations are linked to the heightened expression of genes involved in sterol biosynthesis within human breast tumors.^79^ Mutations in the P53 gene are reportedly associated with aggressive histology and increased proliferation rates in BC. Importantly, compelling evidence establishes a correlation between P53 mutations, lipid metabolism, and tumor architecture.^80^ P53 plays a vital role in lipid metabolism by either directly regulating the expression of genes involved in this process or interacting with key metabolic enzymes. Specifically, mutant P53 has been shown to enhance lipid anabolism.^66^^,^^81^

HRAS, the first human oncogene to be cloned, plays a critical role in oncogenesis. Aberrant activation of the Ras/MAPK pathway is of great significance in cancer initiation and progression and has remained a focal point in cancer research for years. Carcinogenic mutations in the Ras and Raf genes are frequently observed in various cancer types, including lung cancer, colorectal cancer, pancreatic cancer, and melanoma.^82^ Under the influence of growth factors or activated RAS, cells activate mTORC1 through diverse signaling pathways, such as PI3K/AKT and RAF/MEK/ERK. This induces mature SREBP to enter and accumulate in the nucleus, promoting the transcription of genes related to lipid metabolism. Consequently, intracellular lipid synthesis or extracellular fatty acid utilization is enhanced to meet the metabolic demands of tumor cell growth.^83^^,^^84^ KRAS mutations are relatively infrequent in BC. It is speculated that in TBNC, mammary-origin cells employ alternative parallel or redundant signaling pathways to acquire the functions of Ras in lipid metabolism, metastasis, and drug resistance.^85^

#### Extrinsic factors of heterogeneity

**Figure 2:**
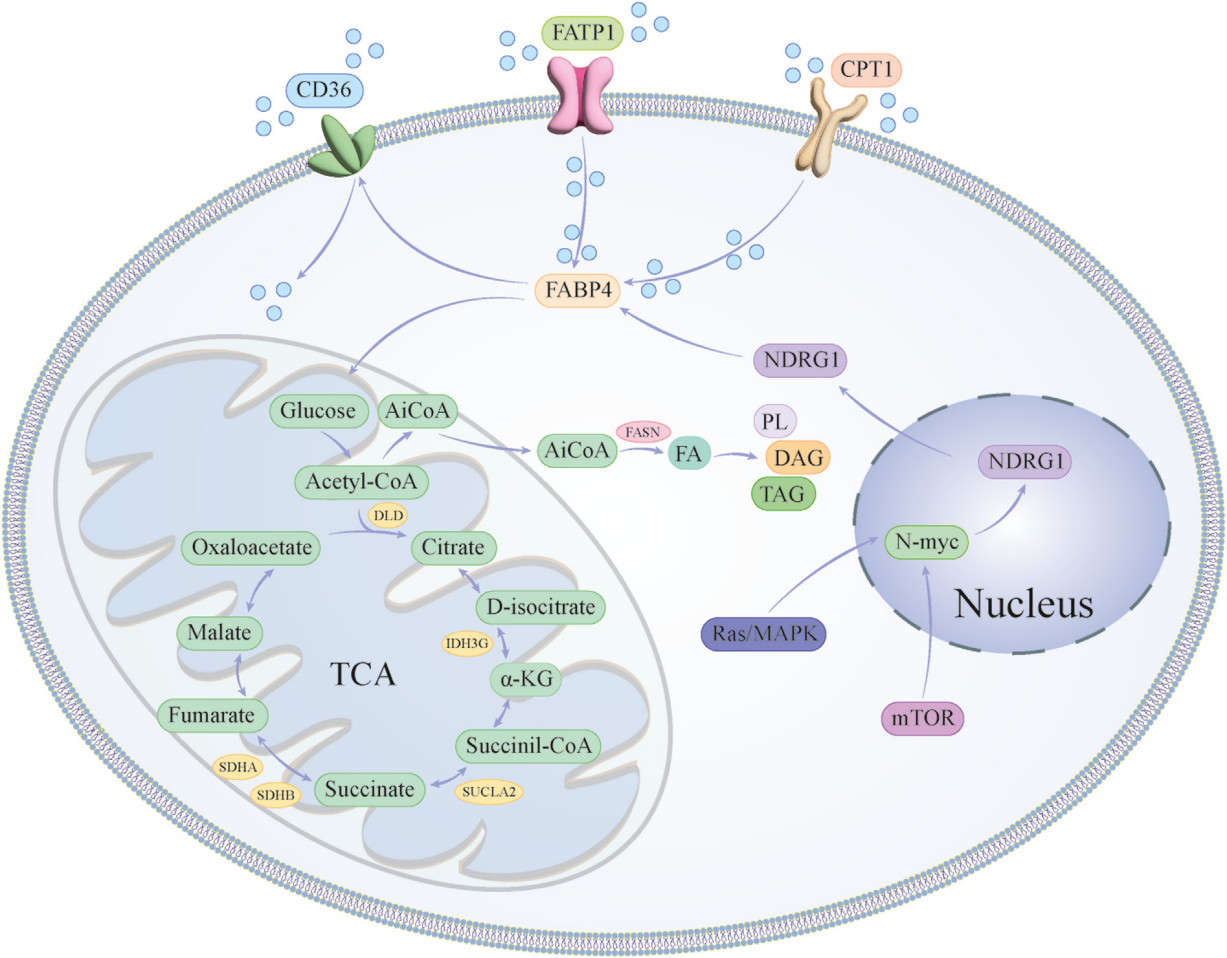
Reconstruction of fatty acid metabolism in breast cancer (BC). This process is influenced by various intrinsic factors, including mutations in P53, Ras, and Raf genes, as well as by extrinsic factors, such as cancer-associated adipocytes (CAA), cancer-associated fibroblasts (CAF), tumor-associated macrophages (TAM), and tumor-infiltrating lymphocytes (TIL). Besides, transmembrane proteins, such as CD36, fatty acid-binding proteins (FABP), and carnitine palmitoyltransferase 1 (CPT1), play a crucial role in regulating fatty acid uptake and metabolism. These factors collectively impact BC cell proliferation, metastasis, and drug resistance.

Cell-extrinsic factors contributing to metabolic heterogeneity encompass interactions with the tissue microenvironment and nutrient availability. Recent literature highlights the metabolic symbiosis between cancer cells and stromal cells as the central mechanism underlying drug resistance, responsiveness to hypoxia, and metabolic stress.^86^, ^87^, ^88^ The dynamic metabolic crosstalk involves various cellular components, such as cancer-associated adipocytes, cancer-associated fibroblasts, tumor-associated macrophages, and tumor-infiltrating lymphocytes, which collectively influence the phenotypic characteristics and fate of tumor cells. In the context of BC, adipocytes present in the breast tissue undergo distinct phenotypic and functional alterations, giving rise to tumor-modified adipocytes, such as cancer-associated adipocytes. Cancer-associated adipocytes induce heightened catabolic processes, resulting in the release of diverse metabolites, including lactic acids, pyruvic acids, and free fatty acids.^89^ The close interaction between cancer-associated adipocytes and BC cells throughout tumor progression seemingly contributes to the invasive properties of tumor cells. The uptake of free fatty acids by BC cells is facilitated by various proteins, such as CD36, FABPs, and carnitine palmitoyltransferase 1 (CPT1), which have implications for tumor progression and invasiveness.^90^ Notably, the levels of reactive oxygen species generated by BC cells exhibit a significant correlation with the characteristics of invasive tumors.^91^ Furthermore, FABP4 secreted by cancer-associated adipocytes has been found to stimulate the expression of multiple fatty acid transporters and promote tumor cell proliferation^92^ (Fig. 2).

### Potential therapeutic targets for lipid metabolism in BC

Cancer cells' lipid metabolism requirements primarily arise from *de novo* fatty acid synthesis^93^^,^^94^ and lipid uptake/storage.^60^^,^^61^ In the new fatty acid synthesis, glucose undergoes glycolysis and the tricarboxylic acid cycle to generate acetyl-CoA and/or citrate.^95^^,^^96^ Further, glutamine is metabolized within mitochondria through the tricarboxylic acid cycle, contributing to fatty acid synthesis.^97^ ATP-citrate lyase facilitates the conversion of citrate to acetyl-CoA, and its overexpression stimulates the proliferation of BC cells. Moreover, it directly inhibits AMPK activity and indirectly inhibits P53 expression, impeding cell senescence and promoting fatty acid synthesis.^98^^,^^99^ FASN, a key enzyme in many cancers, positively correlates with cancer progression, drug resistance, and HER2 positivity rate in patients with BC.^100^ The absorption of exogenous palmitic acid reportedly protects BC cells from the pro-apoptotic effects of FASN inhibition.^101^ FABP4 facilitates fatty acid transport to various cellular compartments, exerting diverse metabolic functions. FABP4 has been found to up-regulate the expression of the fatty acid transport proteins CD36 and FABP5, promoting BC cell proliferation.^92^ N-myc downstream-regulated gene 1 (NDRG1) is overexpressed in BC and is associated with increased metastasis rates and patient mortality by regulating lipid metabolism. Inhibiting NDRG1 expression can reduce cell proliferation rates, presenting a potential therapeutic strategy for patients with BC.^102^ Lipid-associated macrophages constitute a novel subset of macrophages within the tumor-adipose microenvironment, characterized by lipid accumulation and enhanced phagocytic activity. Depleting lipid-associated macrophages in this microenvironment has been demonstrated to synergize with anti-programmed death-1 (PD1) therapy, enhancing anti-tumor effects in allograft cancer mouse models.^103^ The expression of lysine demethylase 5B positively correlates with BC metastasis. Silencing lysine demethylase 5B or employing its inhibitor AS-8351 activates the AMPK signaling pathway, consequently inhibiting BC cell proliferation and migration, offering a new potential therapeutic approach for BC treatment.^104^

### Main pathways of glucose metabolism in organisms and cancer cells

Glucose metabolism is a vital physiological process in organisms, encompassing glucose synthesis and breakdown. The principal metabolic pathways of glucose include glycolysis, gluconeogenesis, glycogen synthesis and breakdown, and tricarboxylic acid cycle.

i) Glycolysis, the primary pathway of glucose metabolism, predominantly occurs in the cytoplasm. During this anaerobic process, glucose is broken down into two molecules of pyruvic acid, generating a specific amount of energy. ii) In gluconeogenesis, an aerobic process, organisms produce glucose by breaking down non-sugar substrates, such as fats and proteins, when glucose is lacking. iii) Glycogen, a polysaccharide, is primarily stored in the liver and muscles. When glucose is required, glycogen is broken down into glucose. Conversely, when there is an excess of glucose, it can be synthesized into glycogen for storage. iv) The tricarboxylic acid cycle, occurring primarily in the mitochondria, represents the final stage of glucose metabolism. In this process, pyruvic acid is completely oxidized, which produces a significant amount of energy.

### Glucose metabolism in cancer

Normal differentiated cells typically rely on mitochondrial oxidative phosphorylation to generate energy necessary for physiological activities. In contrast, many cancer cells exhibit a phenomenon known as the “Warburg effect”, relying on aerobic glycolysis for their energy demands. Although aerobic glycolysis is a less efficient method to produce ATP, it favors the formation of substances crucial for cancer cell proliferation, such as nucleotides, amino acids, and lipids.^105^ Furthermore, BC cells utilize folate and acetate to expedite lipid biosynthesis, which protects them from apoptosis and elevated levels of reactive oxygen species. Consequently, the remodeling of cancer cell metabolism is a significant feature of cancers, including BC.^106^^,^^107^ Rapidly proliferating tumor cells have elevated energy requirements, and sustained hyperglycemia provides a nutritional foundation that promotes malignant tumor cell proliferation and growth.

### The factors affecting glucose metabolism in BC

BC is characterized by metabolic alterations, particularly a pronounced reliance on glycolysis and the occurrence of gene mutations associated with the Warburg effect. Despite sufficient oxygen availability, cancer cells, including BC cells, maintain a preference for glycolysis. Wild-type P53 promotes aerobic respiration and suppresses glycolysis by regulating the expression of P53-induced glycolysis and apoptosis regulator.^108^ However, in BC cells, particularly in basal-like and HER2-enriched subtypes, P53 gene mutations induce increased glycolytic activity.^109^, ^110^, ^111^ Basal-like BC cells, in particular, exhibit significantly elevated c-Myc expression, which contributes to the activation of the aerobic glycolysis pathway.^112^ Mutations in the phosphatidylinositol 3-kinase (PI3K) gene also stimulate glycolysis via the PI3K/Akt/mTOR signaling pathway.^113^ This metabolic rewiring, known as the Warburg effect or aerobic glycolysis, enhances cancer cell proliferation and metastasis.^114^ The genes encoding glucose transporters (GLUTs) and glycolytic enzymes play critical roles in driving the Warburg effect.

### GLUTs, glycolytic enzymes, and their roles in BC

GLUTs, a class of transmembrane proteins, facilitate glucose transportation into cells. There are two types of GLUTs: sodium-dependent GLUTs, which actively transport glucose against its concentration gradient, and facilitative GLUTs, which transport glucose along the concentration gradient without energy consumption. These transporters are present on the cell membranes of various mammalian species. The GLUT family comprises 14 distinct proteins, each exhibiting a specific tissue distribution and regulatory mechanism. There has been particular interest in studying the role of GLUTs in BC. Malignant cells, including BC cells, heavily rely on glucose as their primary source of energy for growth and proliferation. Consequently, cancer cells often augment the expression of GLUTs to enhance glucose uptake.^115^ Increased GLUT1 and GLUT3 expression levels have been observed in BC. The transcriptional co-regulator RIP140, which inhibits the transcriptional activity of the hypoxia-inducible factor HIF-2α, interacts with p53 to suppress GLUT3 gene transcription. By acting as a key regulator of p53/HIF crosstalk, RIP140 inhibits glycolysis and impedes BC cell proliferation.^116^ Moreover, GLUT3 expression is evidently elevated in TNBC and is crucial for promoting epithelial–mesenchymal transition, enhancing invasiveness, and facilitating distant metastasis in TNBC cells.^117^ In the BC cell line ZR-75, the cAMP signaling pathway has been shown to regulate GLUT3 expression and glucose uptake.^118^ Importantly, high expression levels of GLUT1 in BC have been associated with tumor subtype, higher grade, and poor prognosis.^119^ TNBC is also associated with increased expression levels of GLUT1, encoded by SLC2A1, and inhibiting GLUT1 can evidently prevent the growth of RB1^+^ TNBC.^120^ Furthermore, there is evidence of up-regulated expression of sodium-dependent GLUT1 in various cancer types, contributing to cell growth in TNBC.^121^, ^122^, ^123^ Notably, the levels of glycolytic enzymes play a critical role in BC. The pyruvate kinase M2 isoform, a key glycolytic enzyme, undergoes methylation by coactivator-associated arginine methyltransferase 1 (CARM1), which results in the activation of aerobic glycolysis, promoting the development of breast tumors.^124^ Lactate dehydrogenase A, another glycolytic enzyme, is commonly overexpressed in cancer, driving glycolysis and cancer initiation.^125^ Hexokinase 2, also a glycolytic enzyme, promotes the expression of programmed cell death ligand 1 (PD-L1) in human BC specimens, which facilitates immune evasion in BC cells. Therefore, interventions targeting the protein kinase activity of hexokinase 2 hold potential as a treatment strategy for BC.^126^ Phosphoglycerate mutase 1 (PGAM1), a key glycolytic enzyme, has been implicated in tumor progression and metastasis, and its expression is up-regulated in patients with BC. PGAM1 negatively regulates the expression of argininosuccinate synthase 1 (ASS1) through the cAMP/AMPK/CEBPB axis, and its overexpression is associated with poor patient prognosis.^127^ Overall, the dysregulation of the glycolytic pathway plays a crucial role in the malignant development and growth of BC cells.

### Other glucose metabolism pathways and their impact on BC

**Figure 3:**
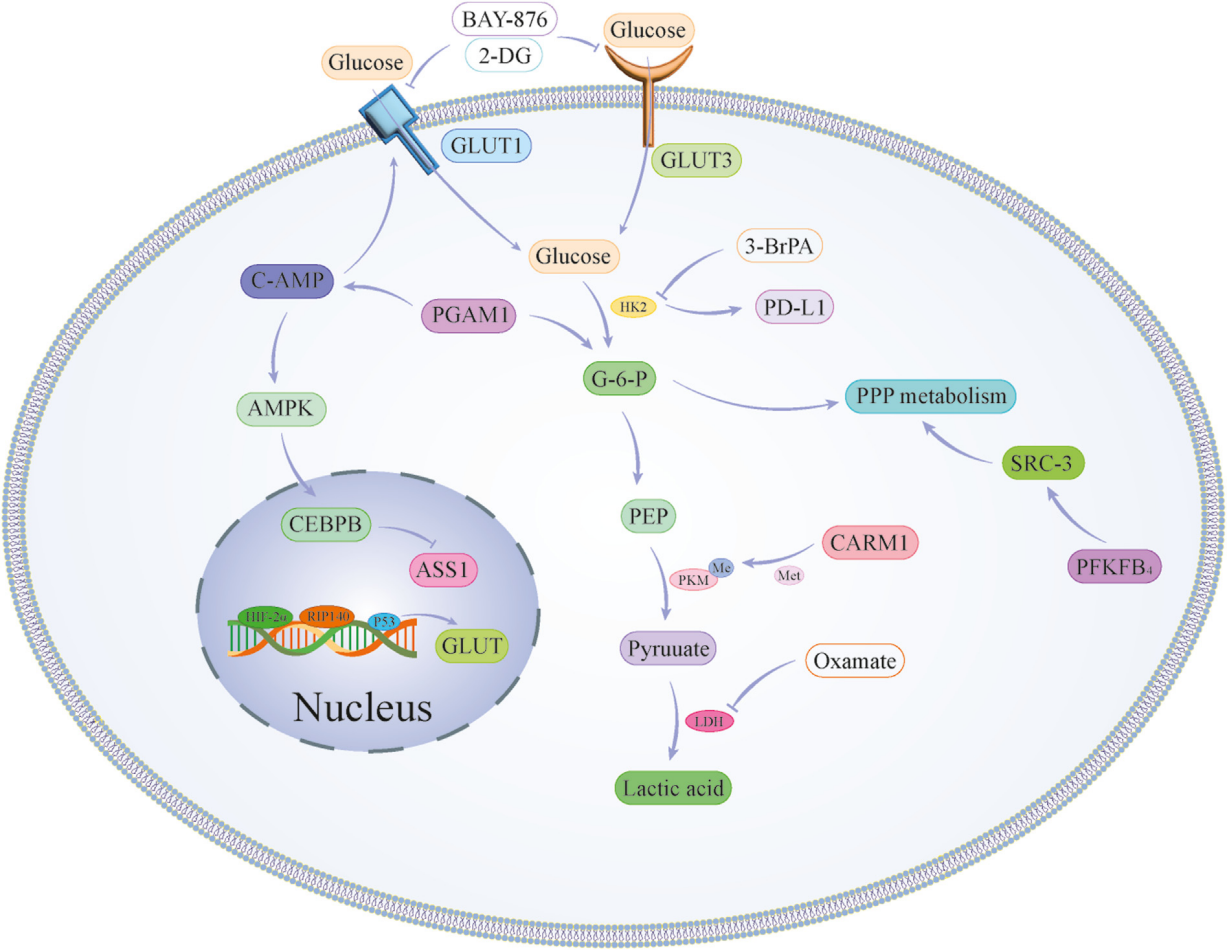
Reconstruction of glucose metabolism in breast cancer (BC). This figure depicts the reconstruction of glucose metabolism in BC, highlighting the pivotal role of genes such as P53, PI3K, and GLUT3 in promoting the Warburg effect within BC cells. These genes regulate enzyme activity and expression in the glucose metabolism pathway, as well as influence the function of glucose transporter proteins (GLUT), significantly impacting BC cell proliferation and metastasis.

The influence of other glucose metabolism pathways on the occurrence and progression of BC is also significant. The metabolic enzyme 6-phosphofructo-2-kinase/fructose-2,6-bisphosphatase 4 plays a crucial role in transcriptional reprogramming by activating oncogenic steroid receptor coactivator-3. This activation shifts glucose metabolism toward the pentose phosphate pathway, triggering purine synthesis through the transcriptional up-regulation of transketolase. Consequently, BC progression is facilitated.^128^ Another pathway of interest is the Rac1-mediated pathway, which activates aldolase A and ERK signaling. This activation leads to the up-regulation of glycolysis, particularly through the non-oxidative pentose phosphate pathway. Consequently, chemoresistance is induced in BC.^129^ Furthermore, the initiation of gluconeogenesis, a central glucose metabolism pathway, is governed by the cytoplasmic enzyme phosphoenolpyruvate carboxykinase. This enzyme is induced by hypoxia, mobilizing transcription factors such as the hypoxia-inducible factor HIF-1α and forkhead box O1 (FoxO1). Its up-regulation subsequently triggers a retrograde carbon flow from gluconeogenesis to glycogenesis, glycogenolysis, and the pentose phosphate pathway, promoting the growth of BC cells.^130^ Besides, glucose-6-phosphate dehydrogenase expression levels vary across different BC subtypes and are positively correlated with poor patient prognosis^131^ (Fig. 3).

### The therapeutics targeting glucose metabolism and GLUTs

In BC, therapeutic strategies primarily aim to hinder glucose metabolism and transport. Reducing glucose uptake by BC cells through GLUT inhibition is a key approach, as is interfering with glycolysis by inhibiting the activity of glycolytic enzymes such as hexokinase and lactate dehydrogenase. Certain drugs, such as metformin, exert their effects by inhibiting the mitochondrial respiratory chain complex, reducing ATP production, and ultimately inhibiting glycolysis.

BAY-876, a selective inhibitor of GLUT1, controls metabolic flux by inhibiting glucose uptake. It specifically affects the acetylation of individual histones and synergistically inhibits TNBC growth by inducing cell apoptosis,^132^ while 2-deoxy-d-glucose, a laboratory-synthesized glucose analog that cannot be metabolized, competes with glucose for binding to GLUTs. This competition reduces glucose intake by BC cells, thereby inhibiting their growth and reproduction.^133^ Both BAY-876 and 2-deoxy-d-glucose reportedly decrease glucose uptake specifically in TNBC cell lines, suggesting potential therapeutic implications. Peptide-based proteolysis-targeting chimeras have shown promise in biomedical applications. Forkhead box M1 (FOXM1), a proliferation-associated transcription factor, is highly expressed in various cancers. A peptide-based FOXM1 proteolysis-targeting chimera was reported to effectively enter cells and induce FOXM1 protein degradation, strongly inhibiting the viability, migration, and invasion of various cancer cell lines, reducing the expression levels of GLUT1 and the immune checkpoint PD-L1, and consequently decreasing glucose metabolism in cancer cells. Therefore, it could be a safe and promising agent for BC treatment.^134^ Studies have indicated that combining GLUT1 inhibitors with cisplatin enhances DNA damage effects and modulates the Akt/mTOR and MAPK signaling pathways, inhibiting the growth of BC cells.^135^ Polyphenols, a type of secondary metabolite in plants, have also demonstrated potential as GLUT1 inhibitors.^136^ They exert hypoglycemic effects by modulating intracellular insulin signaling pathways and inhibiting intestinal enzymes and transporters. Moreover, polyphenols have shown therapeutic effects in various cancers, including BC.^137^ Resveratrol, hesperetin, quercetin, epigallocatechin-3-gallate, and cantharidin are all examples of polyphenols that can inhibit GLUT1 mRNA and protein levels in cancer cells, leading to reduced glucose uptake and inhibition of cancer cell metastasis.^138^, ^139^, ^140^, ^141^ Current research on breast tumors suggests that the up-regulation of GLUT4 expression to induce aerobic glycolysis contributes to drug resistance in targeted cancer therapy. A study found that the N^6^-methyladenosine demethylase ALKBH5 promoted the N^6^-methyladenosine demethylation of GLUT4 mRNA, increasing GLUT4 mRNA stability in a YTHDF2-dependent manner and enhancing glycolysis in drug-resistant BC cells. In cancer tissues from patients exhibiting a poor response to HER2-targeted treatment, an increase in the expression of ALKBH5 or GLUT4 was observed, which was significantly associated with poor prognosis. The ALKBH5-mediated N^6^-methyladenosine demethylation of GLUT4 mRNA promotes resistance to HER2-targeted treatment, suggesting that targeting the ALKBH5/GLUT4 axis has therapeutic potential for treating patients with refractory BC.^142^ Targeting GLUTs as a therapeutic strategy presents several challenges. First, GLUTs are ubiquitously expressed in all tissues, making it difficult to achieve selective inhibition in cancer cells. Second, there are multiple isoforms of GLUTs, and different cancer types tend to express different GLUT isoforms, complicating the development of effective inhibitors. Finally, inhibiting the expression of GLUTs could lead to unwanted side effects due to their essential role in normal physiological processes, such as glucose homeostasis.

Inhibitors that target specific enzymes in glucose metabolism have also shown promise. For instance, 3-bromopyruvate, an inhibitor of hexokinase, induces apoptosis in MDA-MB-231 BC cells.^143^ Methyl jasmonate, another hexokinase inhibitor, reduces tumor volume in mice bearing 4T1 BC cells.^144^ Oxamate, a lactate dehydrogenase inhibitor, has been found to effectively inhibit tumor growth in xenograft models of human MDA-MB-231 TNBC cells.^145^ Galloflavin, an inhibitor of lactate dehydrogenase A, evidently induces cell death in both MDA-MB-231 TNBC cells and tamoxifen-resistant MCF-7 BC cells.^146^ In addition, gossypol, a liposoluble polyphenolic compound, inhibits tumors by targeting lactate dehydrogenase isoenzymes and inhibiting glycolysis.^147^ While these compounds represent some common inhibitors of glucose metabolism enzymes, ongoing research and extensive data are essential for broader application and validation.

## Conclusions

In summary, herein we highlight the common metabolic alterations in BC and the factors influencing these changes. Further, we shed light on potential therapeutic targets and treatment strategies within the metabolic context of BC. BC displays heightened energy metabolism and an increased demand for glutamine. The intrinsic production of glutamine is insufficient to sustain rapid proliferation, necessitating uptake from extracellular sources via membrane transport proteins or up-regulation of key metabolic enzymes in the glutamine metabolism pathway. Lipid metabolism is closely associated with BC metastasis and drug resistance. Lipids serve as an energy source for tumor cells and signaling molecules for intercellular communication. BC cells also exhibit the Warburg effect, relying on glycolysis to generate energy and produce lactic acids to support their growth and shape the tumor microenvironment. The remodeling of metabolic pathways is a crucial aspect of BC as well as other types of cancers. Metabolic treatments for BC have been widely investigated and involve targeting different pathways to hinder tumor growth. However, the clinical outcomes of such approaches often fall short of the anticipated theoretical benefits. Several factors contribute to this discrepancy. First, although theoretical models assume high efficiency and specificity in target inhibition, real-world drug usage frequently fails to achieve optimal efficacy. Second, theoretical mechanisms predominantly explain specific targets regulated by genetic means, while drug actions are often more complex and may involve interactions with other components, leading to alterations in drug efficacy. Lastly, differences between drug administration methods and metabolic stability during treatment can diverge from theoretical research. To address these complexities, multidimensional approaches, including the intersection of metabolism with immunity, must be explored to develop more effective BC treatment strategies for the benefit of patients.

## Funding

The present study was funded by the National Science Foundation for Young Scientists of China (No. 81802665), the Excellent Youth Talent Pool of Army Medical University (China) (No. XZ-2019-505-011), the National Science Foundation for Young Scientists of China (No. 82203057), the Chongqing Major Medical Research Program (Joint Program of Chongqing Municipal Health Commission and Science and Technology Bureau of China) (No. 2024DBXM001), the Chongqing Clinical Diagnosis and Treatment Center of Breast Cancer (China) (No. 425Z2a1), and the Military Key Clinical Specialty (China) (No. 51561Z23612).

## CRediT authorship contribution statement

**Xiujuan Wu:** Funding acquisition, Methodology, Writing – original draft, Writing – review & editing. **Xuanni Tan:** Data curation, Methodology. **Yangqiu Bao:** Data curation, Methodology, Software. **Wenting Yan:** Data curation, Investigation, Resources, Writing – review & editing. **Yi Zhang:** Project administration, Resources, Supervision, Writing – original draft, Writing – review & editing.

## Conflict of interests

The authors have no competing interests to declare.
